# Enzymatic Low Volume Passive Sweat Based Assays for Multi-Biomarker Detection

**DOI:** 10.3390/bios9010013

**Published:** 2019-01-16

**Authors:** Ashlesha Bhide, Sarah Cheeran, Sriram Muthukumar, Shalini Prasad

**Affiliations:** 1 Department of Bioengineering, University of Texas at Dallas, 800 West Campbell Road, Richardson, TX 75080, USA; Ashlesha.Bhide@utdallas.edu (A.B.); Sarah.Cheeran@utdallas.edu (S.C.); 2 Enlisense LLC, 1813 Audubon Pond way, Allen, TX 75013, USA; Sriram.Muthukumar@utdallas.edu

**Keywords:** wearable biosensing, enzyme-based assay, alcohol detection, glucose detection, lactate detection, chronoamperometry, sweat sensing, continuous monitoring

## Abstract

Simultaneous detection of correlated multi-biomarkers on a single low-cost platform in ultra-low fluid volumes with robustness is in growing demand for the development of wearable diagnostics. A non-faradaic biosensor for the simultaneous detection of alcohol, glucose, and lactate utilizing low volumes (1–5 μL) of sweat is demonstrated. Biosensing is implemented using nanotextured ZnO films integrated on a flexible porous membrane to achieve enhanced sensor performance. The ZnO sensing region is functionalized with enzymes specific for the detection of alcohol, glucose, and lactate in the ranges encompassing their physiologically relevant levels. A non-faradaic chronoamperometry technique is used to measure the current changes associated with interactions of the target biomarkers with their specific enzyme. The specificity performance of the biosensing platform was established in the presence of cortisol as the non-specific molecule. Biosensing performance of the platform in a continuous mode performed over a 1.5-h duration showed a stable current response to cumulative lifestyle biomarker concentrations with capability to distinguish reliably between low, mid, and high concentration ranges of alcohol (0.1, 25, 100 mg/dL), glucose (0.1, 10, 50 mg/dL), and lactate (1, 50, 100 mM). The low detection limits and a broader dynamic range for the lifestyle biomarker detection are quantified in this research demonstrating its suitability for translation into a wearable device.

## 1. Introduction

The market for wearable diagnostic devices is projected to rapidly ascend by 23 percent yearly to over $100 billion by 2023 and exceed $150 billion by 2026 [1]. Wearables enable users to receive personalized health data on a range of medical parameters utilizing an approach that non-invasively and seamlessly acquires data on specific digital biomarkers to monitor parameters such as physical activity and heart rate [2]. This wearable technology allows for users to directly obtain information regarding their own bodies and, subsequently, be able to act accordingly, thus permitting for self-diagnosis, predictive preventive care, and management of health conditions [3]. While there exist numerous wearable devices that track digital biomarkers, wearable diagnostics that analyze and monitor biochemical markers are not as available [4]. For instance, to really delve into the status of human health it is necessary to scrutinize human biofluids that can delineate the body’s physiological state. Human sweat is one such biological fluid that contains valuable medical information pertaining to the human health status [5]. Moreover, it is a preferred candidate over other biological fluids due to its ease of access and allows for non-invasive analysis of samples. Correlation between blood lifestyle biomarkers and sweat biomarkers have been established in the research space [6,7,8]. For this reason, sweat based biosensing is vital in detecting specific biological factors that will provide more of an in-depth analysis on the health status of the body. Multiplexed enzyme based-detection of analytes on a single platform remains a daunting challenge in sweat-based detection. However, several researchers have developed enzyme based-biosensing platforms that can quantify and report alcohol, glucose, and lactate levels in biofluids [9,10,11,12,13,14,15]. This paper will focus on demonstrating an enzyme-based biosensing platform for multiplexed detection of alcohol, glucose, and lactate (lifestyle biomarker triad) in human sweat.

Continuous, real-time monitoring of the lifestyle biomarkers triad is imperative as the dysregulation of one of the biomarkers could potentially affect the functioning of the other biomarkers. Diabetes is associated with the inability of the body to produce insulin resulting in abnormal glucose levels in the body. Management of diabetes requires individuals to tightly monitor their blood glucose levels in a continuous manner to minimize health risks. Self-monitoring of diabetes allows individuals to keep track of their lifestyle and take appropriate measures to keep a control on their glucose levels. Literature studies have revealed a U-shaped physiological connection between alcohol consumption and diabetes [16]. Moderate alcohol consumption by diabetic and pre-diabetic populations affects the glycemic index of the individual depending on their nutrition states causing acute hypoglycemia in fed states and hyperglycemia in unfed states [17]. Excess consumption of alcohol also leads to alcohol ketoacidosis which is potentially fatal in starved conditions. Studies also reveal a relation between blood glucose and blood lactate levels wherein incidence of type 2 diabetes is associated with high plasma lactate levels [18]. This condition is known as lactate acidosis which is related to increased lactate production in diabetic individuals causing a pH imbalance in the body [19]. The correlation between the biomarkers justifies the need to develop a non-invasive multi-biomarker platform that would allow diabetic and pre-diabetic cohorts to self-monitor physiological parameters for diabetes and lifestyle management. This work is a novel demonstration of a sweat-based multi-biomarker detection platform developed on a flexible substrate for non-invasive analysis of lifestyle biomarkers in low volumes of sweat. The nanoporosity of the polyamide substrate aids in uniform fluid transport and enhances the charge storage capacity which is leveraged in this research for enhanced sensitivity and wider dynamic range of biomarker detection. Biosensing is achieved by a novel electrode stack which employs nanotextured zinc oxide thin films as the active biosensing region which allows for increased binding of proteins to the surface for enhanced sensitivity and provides biocompatibility for wearable applications. The biomolecular events occurring at the electrode–sweat interface are captured as capacitive current changes through non-faradaic chronoamperometry (CA). The sensor performance metrics—limit of detection, dynamic range, signal-noise threshold, and specificity of biomarker detection are reported. The stable operation of the biosensing platform over a 1.5-h duration across the established dynamic range on continuous exposure to sweat biomarkers is demonstrated.

## 2. Materials and Methods

### 2.1. Materials and Reagents

Polyamide substrates with a pore size of 200 nm and a thickness of 60 µm were obtained from GE Healthcare Life Sciences (Piscataway, NJ, USA). The linker molecule dithiobis succinimidyl propionate (DSP), dimethyl sulfoxide (DMSO), and 1X phosphate buffered saline (PBS) were procured from Thermo Fisher Scientific Inc. (Waltham, MA, USA). Salt-free streptavidin from Streptomyces avidiini (≥13 units/mg protein), alcohol oxidase enzyme from Pichia pastoris (10–40 units/mg protein), glucose oxidase from Asperigillus niger (100,000–250,000 units/g), D-(+)-glucose, sodium L-lactate (~98% purity), absolute ethyl alcohol (≥99.5%), and sodium bicarbonate (≥99.7%) were procured from Sigma-Aldrich (St. Louis, MO, USA). NHS-biotin was purchased from Vector laboratories (Burlingame, CA, USA). Glucose oxidase antibody was purchased from Abcam (Cambridge, MA, USA). Lactate oxidase (80 U/mg) was purchased from Toyobo USA. Synthetic sweat was prepared from the recipe described in M.T. Mathew et al. [20]. The pH range was varied by varying the concentrations of the constituents. Single donor human sweat of pH~6 was purchased from Lee Biosolutions Inc. (Maryland Heights, MO, USA). No preservatives were added to this product and it was stored at −20 °C. All alcohol, glucose, and lactate dilutions were made in synthetic sweat pH 6, 8, and in human sweat buffers.

### 2.2. Sensor Fabrication

**Figure 1:**
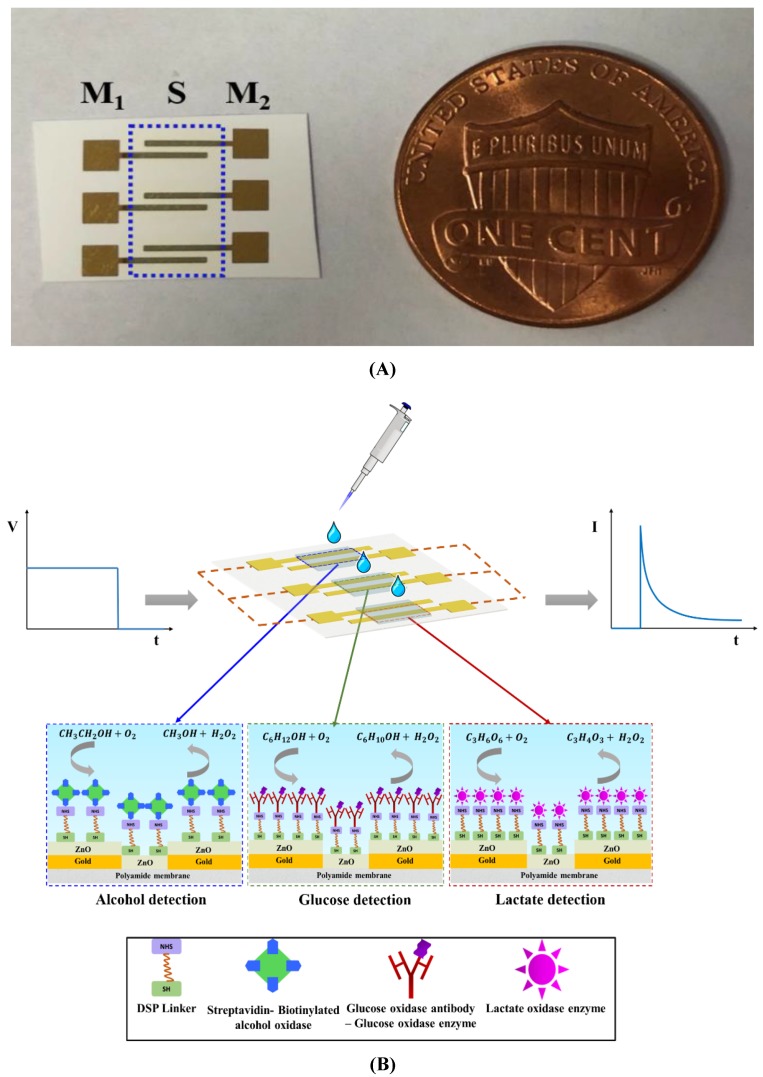
(**A**) Schematic of the enzyme based- biosensing platform (M_1_, M_2_ are the gold measurement electrodes; S is the ZnO active biosensing region); (**B**) Schematic of enzyme complexes functionalized for alcohol, glucose, and lactate detection.

The biosensing platform was deposited on a flexible nanoporous polyamide membrane as shown in Figure 1A. The biosensor comprises of gold measurements (M_1_ and M_2_) electrodes and a ZnO active biosensing region (S). Fabrication of the biosensing platform is a two-step process. Firstly, it involves the deposition of ~150 nm gold electrodes on the substrate using a Temescal e-beam evaporator tool (Ferro Tec, Livermore, CA, USA) and secondly, it involves the sputtering of ZnO thin films in the overlap region between the two gold electrodes using AJA Orion RF magnetron with a 99.999% ZnO target (Kurt J. Lesker) at room temperature. The film thickness is measured using a Veeco Dektak 8 profilometer and is found to be~100–120 nm.

### 2.3. Alcohol Biosensor Calibration in Synthetic Sweat pH 6 and Human Sweat

The enzyme complex immobilized on the alcohol biosensor for the detection of alcohol in synthetic sweat and human sweat is depicted in Figure 1B. The biosensing surface was functionalized with 10 mM DSP thiol-cross linker diluted in dimethylsulfoxide (DMSO) and was dispensed on the ZnO sensing region for 3 h in darkness. Sample volumes were maintained at 3 μL and dispensed on the backside of the active sensing ZnO region all throughout this research. 1 mg/mL of streptavidin in 1X PBS was incubated on the sensing region for 60 min. After immobilizing streptavidin, biotinylated alcohol oxidase enzyme was incubated on the sensing region for 15 min. The enzyme biotinylation process was performed as per the method outlined in Du et al. [21]. Synthetic and human sweat buffers were dispensed on the sensing region depending on the detection buffer. This step was considered as the baseline step. Ethanol was diluted in synthetic sweat buffers in a logarithmically increasing concentration range between 0.01–100 mg/dL. Ethanol dilutions in sweat were dispensed on the sensor in increasing dose concentrations and incubated for 10 min each. CA measurements were performed after every immobilization step. CA measurements were recorded as current measurements using a potentiostat (Gamry Instruments, Warminster, PA, USA) after applying an DC excitation signal of 600 mV for 1-min duration. All data is represented as mean ± relative standard deviation (RSD). A sample set of n = 3 was used throughout this research for building CDRs in synthetic and human sweat buffers.

### 2.4. Glucose Biosensor Calibration in Synthetic Sweat pH 6 and Human Sweat

The enzyme complex immobilized on the ZnO surface for detection of glucose in synthetic and human sweat buffers is shown in Figure 1B. The protocol was adapted and modified based on the protocol published by the group previously [22]. Initially, the biosensing surface is immobilized with 10 mM DSP cross-linker after a 3 h incubation period. The surface is then incubated with 100 μg/mL glucose oxidase antibody for 15 min followed by immobilization of 100 μg/mL glucose oxidase enzyme. Synthetic sweat of pH 6 is dispensed on the sensing region and is considered as the baseline with respect to which all current changes are computed. Glucose dilutions of concentrations from 0.01–50 mg/dL were made in synthetic sweat and human sweat buffer solutions and were applied to the biosensing surface in increasing concentrations to obtain a CDR. A DC bias of 700 mV was applied for 1 min to obtain the current responses.

### 2.5 Lactate Biosensor Calibration in Synthetic Sweat pH 6 and Human Sweat

The lactate detection enzyme complex functionalized on the ZnO surface for lactate detection in synthetic and human sweat buffers is shown in Figure 1B. The DSP functionalized surface is treated with 4 mg/mL of lactate oxidase and incubated for 1.5 h. Lactate-free synthetic sweat was applied to the sensing region to obtain baseline current measurement. Lactate dilutions of concentrations—0.1, 1, 10, 50, 100 mM—were made in synthetic and human sweat buffers. Lactate dose incubation time was maintained at 5 min. Dose–response curves were obtained by applying a DC bias of 650 mV for 1 min.

### 2.6. Specificity Study in Synthetic Sweat pH 6

The specificity study on the biosensing platform was carried out by dispensing 3 μL of cortisol dose concentrations spiked in synthetic sweat pH 6 in increasing dose concentrations. Cortisol dose concentrations in the ranges 0.01–100 mg/dL, 0.01–50 mg/dL, and 0.1–100 mM spiked in synthetic sweat pH 6 were dispensed serially on the alcohol, glucose, and lactate biosensing regions respectively and their current responses were recorded. The incubation times and DC biases were maintained to be the same as required for target biomarker–enzyme interaction as described in the above section. Cortisol free-synthetic sweat pH 6 is considered to be the baseline for all experiments with respect to which all current changes were computed.

### 2.7. Continuous Monitoring in Synthetic Sweat pH 6

Continuous monitoring of alcohol, glucose, and lactate in synthetic sweat pH 6 was performed by dispensing 3 µL of biomarker dose concentrations every 7 min on the ZnO sensing region in succession over a 1.5 h duration. Alcohol concentrations of 0.1, 25, and 100 mg/dL were prepared by spiking alcohol in synthetic sweat pH 6. Each dose concentration was applied to the sensing region five times in progression to obtain the current response for cumulative dosing. The dynamic current response of the biosensing system for continuous alcohol monitoring was measured using the conditions mentioned in the previous section. For continuous glucose and lactate biosensing, glucose, and lactate concentrations of 0.1, 10, 50 mg/dL, and 1, 50, 100 mM respectively were made in synthetic sweat pH 6 and dosed similarly as done for continuous alcohol biosensing. The current responses were plotted against cumulative dose concentrations and the slope changes within each dose regime were computed with respect to time.

## 3. Results and Discussion

### 3.1. Non-Faradaic Chronoamperometry as a Technique for Evaluating the Biosensing Performance

The electrochemical binding events occurring at the ZnO active biosensing region and sweat interface is captured by non-faradaic chronoamperometry. A charged electrode in contact with an electrolyte results in the formation of electrical double layer (EDL) that is equivalent to a capacitance system. EDL is compact region consisting of co-ions (electrode) and counter ions (electrolyte) held together by electrostatic forces of attraction and a diffuse layer. Non-faradaic chronoamperometry is a technique that involves perturbation of the EDL by a step-DC bias which captures the current charge–discharge dynamics of the binding events occurring at the interface. The DC bias input is related to the electroactivity of the biomarker of choice. The output chronoamperometric responses are recorded as time-based current changes. The current response, as shown in Figure 1B, consists of two regions: (1) a current spike arising from the EDL charging, (2) a current decay caused by the EDL relaxation (I_cap_α e^−t/R*C^) reaching a steady state [23]. The catalytic oxidation of the sweat based-lifestyle biomarker concentrations by the enzyme system produces H_2_O_2_ and other products causing a charge redistribution in the existing EDL. The current produced by this charge modulation is captured as the biosensing response of the developed platform.

### 3.2. Fluid Wicking Study and Electrical Characterization of the Surface Functionalized Biosensor

**Figure 2:**
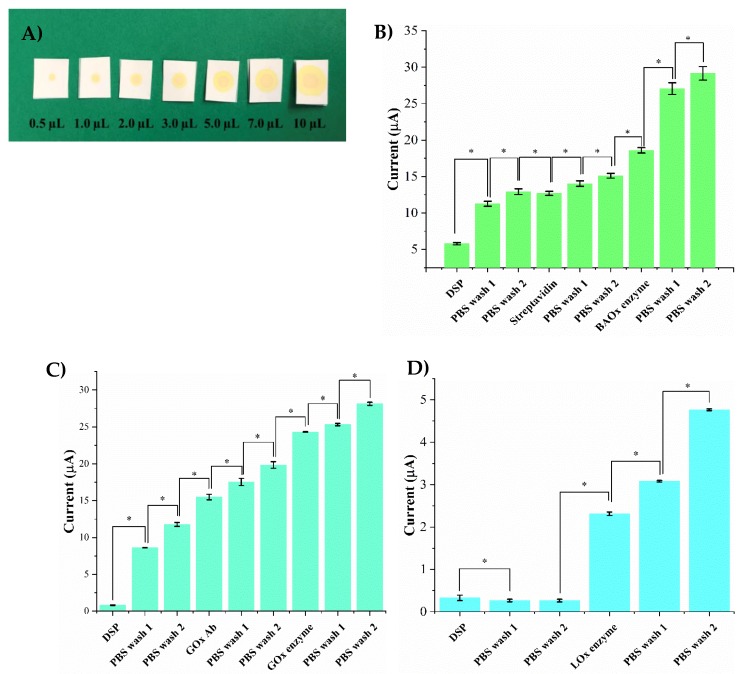
(**A**) Fluid wicking capability of the polyamide substrate for different sweat volumes (0.5–10 μL). Electrical characterization of enzyme complex functionalized on the ZnO surface for (**B**) Alcohol biosensor (**C**) Glucose biosensor (**D**) Lactate biosensor. Statistical significance between each assay step is set at threshold of 0.05 (*p* < 0.05).

In this work, we have utilized a flexible, nanoporous polyamide membrane suitable for wearable applications with a capability of wicking sweat. The porous and the fibrous network of the polymer that mimics a fabric allows for the easy transport of sweat from the skin to the active biosensing ZnO region of the sensor platform. The group has previously characterized the structural and functional utility of the substrate and the ZnO active biosensing region for wearable biosensing applications [9,22,24]. The hydrophilic nature of polyamide allows uniform diffusion of sweat throughout the entire sensing region and requires less incubation time. The wicking profiles of 0.1–10 μL volumes of liquid on the polyamide membrane are shown in Figure 2A. It is evident that the fluid spreads uniformly in all directions and that low-volumes of 1–5 μL are adequate for the robust biosensing of the sweat biomarkers of choice.

Robust biosensing requires the enzyme complexes to be successfully functionalized and confined within the nanopores of the membrane. Current response is used to characterize the chemical interaction occurring between the ZnO biosensing surface and various enzyme complex steps as described in Section 3.1. The first step for immobilizing an enzyme complex on the sensing platform involves incubation of a cross-linker DSP on the biosensing surface for 3 h. The thiol group of the DSP binds to the ZnO surface through Zn-S bonds producing a very low current response as DSP (dissolved in DMSO) is resistive in nature. Subsequently, two PBS washes are performed to remove any unbound DSP and to allow further immobilization indicated by a notable change in current. For the alcohol biosensor, streptavidin is incubated for one hour on the DSP functionalized surface. The NHS group of the DSP binds to the amine group of the streptavidin producing a 6 μA increased change in current from the DSP step as shown in Figure 2B. This increased current can be attributed to the presence of charged molecules on the surface making it more conductive. Further, two PBS washes are performed to remove any unbound streptavidin and current responses are recorded to ensure stable binding. The next step involves the incubation of biotinylated alcohol oxidase enzyme on the streptavidin functionalized surface for 15 min. A current change from 15 μA to 18.5 μA is observed ensuring the successful binding of the enzyme to streptavidin. Furthermore, two PBS washes are performed to remove any unbound enzyme. A minor change in current of 0.8 μA between the PBS wash step and the enzyme step is observed validating the binding of the enzyme to the surface. The current response produced by the control experiment performed to ensure the fidelity of glucose oxidase enzyme binding to the sensor surface is shown in Figure 2C. Post DSP functionalization and PBS washes, glucose oxidase antibody is incubated on the surface for 15 min. A current change of 7 μA is observed from the PBS step to the antibody incubation step confirming the binding of the antibody to NHS ester of the DSP. Thereafter, two PBS washes are performed to wash any unbound antibody. The glucose oxidase enzyme is incubated on the antibody immobilized surface for 15 min which produces a current change of 5 μA from the previous PBS wash step. Two PBS washes are performed after the enzyme step producing a minor change in current of 1 μA from the enzyme immobilization step validating the successful binding of the enzyme complex components to the ZnO surface. The validation of the enzyme complex immobilization for lactate detection is shown in Figure 2D. Lactate oxidase enzyme is incubated on the DSP functionalized surface for 1.5 h. Post DSP-binding, a change in current from 0.2–2 μA is observed from the previous PBS wash step confirming that lactate oxidase is bound to DSP. Two PBS washes are performed afterwards to remove any unbound molecules and to activate the surface which can be explained a minor change in current of 0.7 μA. Statistically significant changes in current were observed for each assay step with a *p*-value < 0.05.

### 3.3. Biosensor Calibration in pH Variant Synthetic Sweat

Under normal homeostasis, the pH of human sweat varies between 4.5–7.0 [25]. A dysregulation in the homeostatic conditions of the human body leads to an acid/base imbalance causing pH variations in sweat. Robust biosensing requires stable operation of the biosensor in varying sweat pH microenvironments and to produce an output that is invariant to fluctuating sweat pH values. From the perspective of translation of the developed platform into a wearable diagnostic, it is essential to conserve the biosensing performance metrics across all pH conditions. Investigations of the active biosensing element Zinc oxide (ZnO) in acidic and basic pH solutions have revealed chemical stability and film durability over prolonged periods of time [26,27]. Hence, it is important to characterize the effect of pH variation on the biosensing response on interaction with synthetic sweat of pH values of 6 and 8.

**Figure 3:**
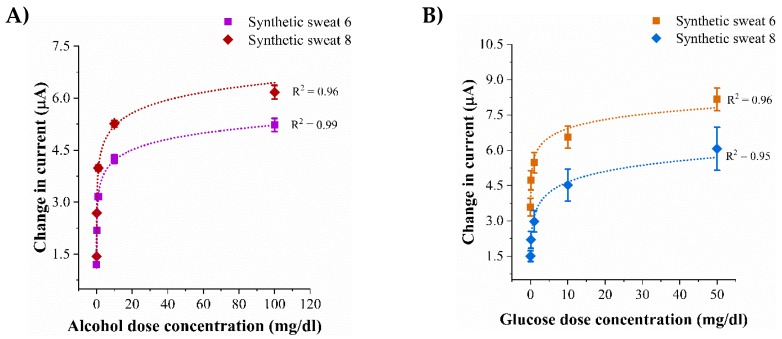

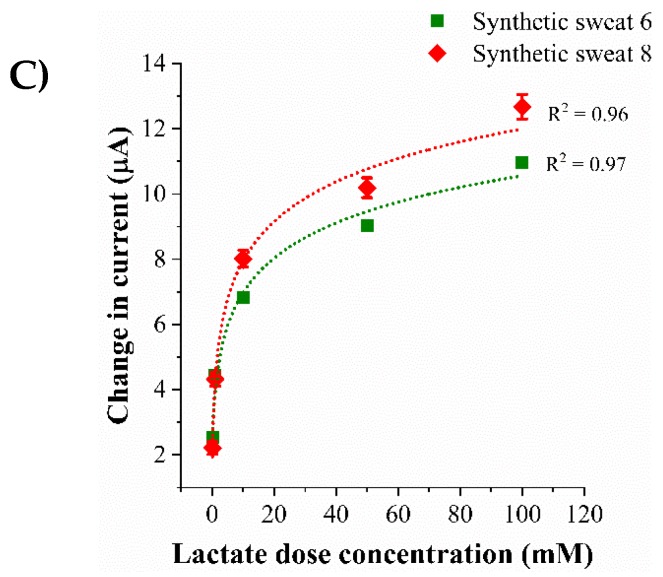
Calibration dose response curves in synthetic sweat pH 6 and 8 for detection of (**A**) Alcohol (**B**) Glucose (**C**) Lactate.

Enzyme based biosensing is demonstrated using non-faradaic chronoamperometry to detect the biomarkers of choice in sweat. The electrochemical response of the lifestyle biomarker triad to increasing dose concentration varying pH sweat solutions is represented as calibration dose response (CDR). The CDR curves are plotted as a function change in steady-state current obtained from a dose concentration with respect to the steady-state current obtained from a zero-dose concentration that does not consist of any molecules of the biomarkers of choice and is termed as the baseline. The dose response of alcohol biosensor to an alcohol concentration range of 0.01–100 mg/dL in sweat pH values of 6 and 8 are shown in Figure 3A. The change in current from low to high alcohol dose in sweat pH 6 is 1.2 (±0.006)–5.2 (±0.036) μA. The current change from low dose to high dose for sweat pH 8 is 1.4 (±0.001)–6.2 (±0.032) μA implying an increasing current being generated with increasing dose concentrations because of the catalytic oxidation reaction occurring between the enzymatic system and the biomarkers of choice. Considering a signal to noise ratio (SNR) of 3, the calculated noise thresholds for sweat pH values of 6 and 8 lie below the current response obtained from the lowest detectable dose concentration of 0.1 mg/dL which is termed as the limit of detection (LOD) [28]. The dynamic range for reliable alcohol detection is 0.1–100 mg/dL. Figure 3B represents the dose responses of varying glucose concentrations 0.01–50 mg/dL spiked in sweat pH’s 6 and 8 on interaction with the glucose biosensor. In sweat pH 6, the change in current from low glucose concentration to high glucose concentration is 3.5 (±0.18)–8.1 (±0.1) μA. The current change observed from low to high glucose dose concentration in sweat pH 8 is 1.5 (±0.24)–6.6 (±0.26) μA. The glucose detection limit is found to be 0.1 mg/dL and the dynamic range is 0.1–50 mg/dL. The dose response for lactate biosensing over a concentration range of 0.1–100 mM in sweat pH values of 6 and 8 are shown in Figure 3C. The current changes for 0.1 mM in sweat pH values of 6 and 8 are 2.5 (±0.003) μA and 2.2 (±0.008) μA. For 100 mM lactate concentration, the change in current in sweat pH values of 6 and 8 are observed to be 10 (±0.007) μA and 2.2 (±0.003) μA. The lowest detection lactate concentration is 0.1mM and the dynamic range of detection is found to be 1–100 mM. Differences of 2–5% in the magnitudes of the current responses between sweat pH 6 and 8 could be due to the excess H^+^ ions participating in the charge transfer reaction occurring between the enzymatic system and the active biosensing ZnO region of the electrode. Similar trends in dose–response curves are produced by the lifestyle biomarkers in sweat pH values of 6 and 8 indicating that pH variation has minimal effect on the output response and does not degrade the biosensing performance.

### 3.4. Biosensor Calibration in Human Eccrine Sweat

**Figure 4:**
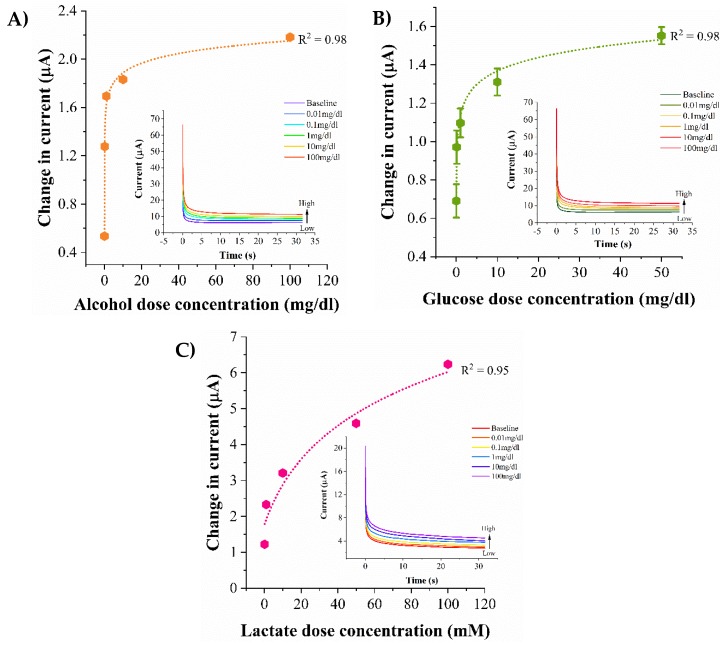
Calibration dose response curves in human eccrine sweat for detection of (**A**) Alcohol (**B**) Glucose (**C**) Lactate. Chronoamperograms obtained from the biosensing regions are shown as figure insets.

Human sweat is a complex biomatrix consisting of electrolytes and metabolites which serve as biomarkers for non-invasive dynamic monitoring of physiological conditions in human body. The current obtained from the biosensing platform in response to the lifestyle biomarkers spiked in human sweat is represented as calibration dose response curves as shown in Figure 4A–C. The pre-existing concentrations of the lifestyle biomarkers present in the human sweat sample procured under normal conditions is considered as the baseline with respect to which the current changes obtained from the dose concentrations are computed. As a consequence of catalytic oxidation reactions occurring at the biosensing interface, increasing concentrations of the lifestyle biomarkers leads to an increased production of H_2_O_2_ and by-products which in turn generate an increasing capacitive current at the interface (see Figure 4 insets). The dose response of the alcohol biosensor to 0.01, 0.1, 1, 10, 100 mg/dL spiked in human sweat is shown in Figure 4A. The current change from the baseline for the alcohol dose concentrations is observed to be 0.5 ± 0.03 μA–2.2 ± 0.007 μA for the lowest and the highest alcohol dose concentrations respectively. The limit of alcohol detection is found to be 0.1 mg/dL and the dynamic range is 0.1–100 mg/dL. Figure 4B represents the calibration curve for glucose concentrations 0.01, 0.1, 1, 10, 50 mg/dL in human sweat. The current changes from the baseline for 0.01 mg/dL and 50 mg/dL are 0.7 ± 0.2 μA and 1.55 ± 0.5 μA. The lowest detectable glucose concentration is 0.1mg/dL and the dynamic range of glucose detection in human sweat is 0.1–50 mg/dL. The dose response for lactate biosensing in human sweat for concentrations 0.1, 1, 10, 50, 100 mM is shown in Figure 4C. The range of current changes obtained for a low dose of 0.1 mM to a high lactate dose concentration of 100 mM is observed to be 1.2 ± 0.002 μA to 6.3 ± 0.04 μA. For an SNR of 3, the LOD and the dynamic range of lactate detection are found to be 1 mM and 1–100 mM respectively. Lower magnitudes of current changes are observed in human sweat in comparison to synthetic sweat buffers owing to the contributions of the interferents present in human sweat to noise threshold of the biosensing system.

### 3.5. Evaluation of Sensor Specificity in Synthetic Sweat pH 6

**Figure 5:**
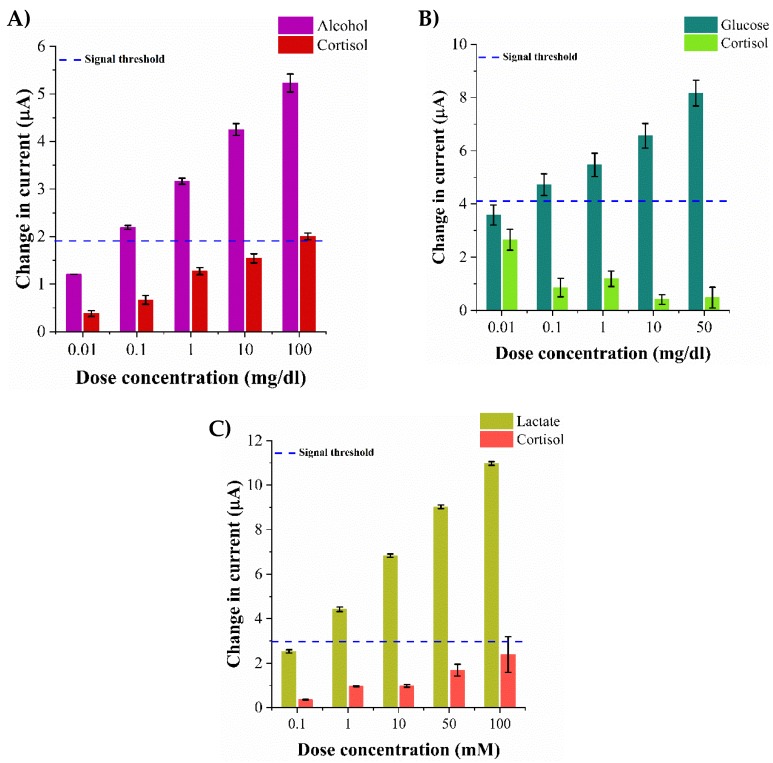
Specificity study with cortisol as the non-specific molecule in synthetic sweat pH 6 for (**A**) Alcohol biosensor (**B**) Glucose biosensor (**C**) Lactate biosensor.

As discussed previously, human sweat consists of other components that interfere with the detection of the lifestyle biomarker specific to its enzyme thus contributing to the electronic noise of the system from the undesired interactions. For the development of robust multi-biomarker detection platforms, it is essential to characterize the cross responses obtained from the interactions occurring between the specific functionalized assay and the non-specific biomarkers. The specificity of the platform is assessed by allowing the individual biosensor to interact with a non-specific sweat-based biomarker. We have evaluated the non-specific responses of the biosensing platform in the presence of cortisol to assess the robustness of the platform in detecting the target biomarkers. The specificity of the biosensing platform in the presence of various cortisol concentrations spiked in synthetic sweat pH 6 is carried as outlined in Section 2.6. The average current change obtained from cortisol on interaction with alcohol oxidase enzyme for the concentration range 0.01–100 mg/dL is 0.3 ± 0.16 μA–2 ± 0.03 μA as shown in Figure 5A. The cross-reactive response obtained from cortisol interacting with glucose oxidase enzyme within the range 0.01–50 mg/dL is 2.6 ± 0.14 μA–0.4 ± 0.08 μA as shown in Figure 5B. Similarly, the current change obtained from the cross-reactive interaction of cortisol with lactate oxidase enzyme in the range 0.1–10mM is 0.4 ± 0.14 μA–2.4 ± 0.33 μA as shown in Figure 5C. The electrochemical current responses obtained from the specific target biomarker–enzyme interactions are ~30% greater than the cross-reactive current reponses obtained from cortisol interaction with the biosensing platform. The non-specific signal obtained from cortisol lies well within the established signal–noise threshold of the system.

### 3.6. Continuous Monitoring of Lifestyle Biomarkers in Synthetic Sweat pH 6

**Figure 6:**
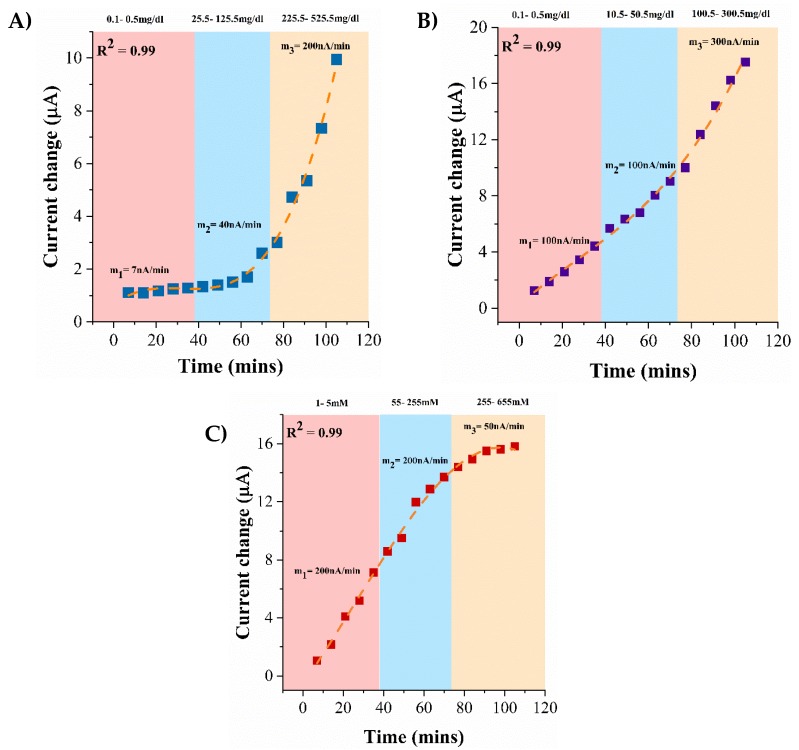
Continuous monitoring of (**A**) Alcohol (**B**) Glucose (**C**) Lactate in synthetic sweat pH 6.

Biosensors can be integrated into a wearable platform by enclosing miniaturized sensors into portable formats with a capability to store data obtained in continuous manner with the intent to periodically provide a feedback to the user for monitoring physiological conditions. The biosensing platform is subjected to continuous dosing of lifestyle biomarkers spiked in synthetic sweat pH 6 over a 1.5-h window as a proof-feasibility for translation into real-time applications. The continuous biosensing profiles captured for alcohol, glucose, and lactate on interaction with the enzyme complexes specific to their detection is shown in Figure 6A–C respectively. With increasing cumulative dose concentrations, the change in the current response from the baseline is found to be incremental. The incremental current changes indicate the responsiveness of the immobilized enzyme complex to incremental biomarker dose concentrations. Slope changes are computed to understand the (1) capability of the biosensing platform in distinguishing between different dose regimes and (2) dynamic interaction of the target biomarker–enzyme complex in real-time. The continuous dose response of cumulative alcohol dose concentrations when dosed continuously in three concentration regimes—0.1, 25, 100 mg/dL—is depicted in Figure 6A. In reach regime, the doses are applied in succession every 7 min. The slope in each regime shows an incremental response with a low current slope of 7 nA/min in the 0.1–0.5 mg/dL regime and a steeper current slope of 200 nA/min in the 225.5–525.5 mg/dL regime. The alcohol biosensor is less sensitive in the lower concentration regime but begins to show a greater current response in the higher concentration regime with minimum signs of saturation. In an analogous manner, the current response of the glucose biosensor when subjected to cumulative dose concentrations in the regimes—0.1, 10, 50 mg/dL—is depicted in Figure 6B. Incremental glucose concentrations produce an incremental current change and thus, an incremental slope change in every regime. The slope of the current change curve from the low to the high regime is 100–300 nA/min. The glucose biosensor is found to be sensitive to consecutive dose concentrations in each regime with an average change in current dose of 4 μA from the low dose to the high dose in the low and mid concentration regime, and 6 μA in the highest concentration regime. The continuous biosensing profile of lactate biosensor to cumulative lactate concentrations in the low, mid, high regimes—1, 50, 100 mM—is represented in Figure 6C. Similar slope changes of 200 nA/min are observed in the low and the mid regimes which is indicative of the lactate biosensor being sensitive in the low and mid concentration regime. However, a low current change slope of 50 nA/min is observed in the higher concentration regime and the slope tapers off which indicates signal saturation. The continuous lifestyle biomarker monitoring study reveals the functionality of the biosensor in all concentrations regimes and can be used for detection of the lifestyle biomarker triad in low volumes of eccrine human sweat.

## 4. Conclusions

In summary, we have demonstrated the development and functionality of a novel, flexible, non-invasive multi-biomarker detection platform suitable for wearable applications. This work outlines the biosensing capabilities of the biomarker detection platform in steady state as well as in a continuous format for up to 1.5 h with minimal signs of signal saturation. We have demonstrated robust detection of alcohol, glucose, and lactate in their physiologically relevant ranges in 1–5 μL sweat volumes on a hybrid metal–metal oxide biosensing platform. Biosensing is achieved by capturing the charge–discharge current responses occurring at the electrode–sweat interface. The fidelity of the enzyme complex binding to the active biosensing region is confirmed by electrochemically characterizing the current responses obtained from the binding of each enzyme complex component. pH studies revealed stable biosensing and the response of the system is preserved in pH variant sweat conditions. The limit of detection for biomarker detection in human sweat was established to be one logarithmic concentration lower and the dynamic range was established to be one logarithmic concentration higher than the physiological relevant range of the biomarkers. The immobilized enzymatic assays specific to each biomarker in the presence of cortisol as the non-specific molecule produced a specific response with minimal cross-talk from the interferents. The developed biosensing platform on integration with portable electronics has the potential to be a self-monitoring wearable device for real-time tracking of human lifestyle.
